# The Homeodomain Transcription Factor Hoxa2 Interacts with and Promotes the Proteasomal Degradation of the E3 Ubiquitin Protein Ligase RCHY1

**DOI:** 10.1371/journal.pone.0080387

**Published:** 2013-11-07

**Authors:** Isabelle Bergiers, Laure Bridoux, Nathan Nguyen, Jean-Claude Twizere, René Rezsöhazy

**Affiliations:** 1 Molecular and Cellular Animal Embryology Group, Life Sciences Institute, Université catholique de Louvain, Louvain-la-Neuve, Belgium; 2 Laboratory of Signaling and Protein Interactions, GIGA-R, University of Liege, Liège, Belgium; Cardiff University, United Kingdom

## Abstract

Hox proteins are conserved homeodomain transcription factors known to be crucial regulators of animal development. As transcription factors, the functions and modes of action (co-factors, target genes) of Hox proteins have been very well studied in a multitude of animal models. However, a handful of reports established that Hox proteins may display molecular activities distinct from gene transcription regulation. Here, we reveal that Hoxa2 interacts with 20S proteasome subunits and RCHY1 (also known as PIRH2), an E3 ubiquitin ligase that targets p53 for degradation. We further show that Hoxa2 promotes proteasome-dependent degradation of RCHY1 in an ubiquitin-independent manner. Correlatively, Hoxa2 alters the RCHY1-mediated ubiquitination of p53 and promotes p53 stabilization. Together, our data establish that Hoxa2 can regulate the proteasomal degradation of RCHY1 and stabilization of p53.

## Introduction

*Hoxa2* belongs to the mammalian *Hox* genes family that encodes 39 highly conserved homeodomain transcription factors mainly involved in embryonic development but also in a large number of pathological processes [1-4]. The developmental roles of Hox proteins have been extensively studied over the past decades and revealed to cover axial patterning of the embryo [5,6], limb formation [7,8], organogenesis processes [9-11] or haematopoiesis [12,13].

At the molecular level, the transcriptional activities of Hox proteins have been well documented and a handful of Hox transcriptional cofactors could be identified, such as Pbx or Meis proteins that belong to the TALE proteins family [14,15]. Hox transcriptional targets were also functionally identified through transcriptomic screenings [16,17] and chromatin immunoprecipitation assays [18-21].

In parallel to their transcriptional activity, some Hox proteins have been involved in non-transcriptional processes. First, Hox proteins have been associated with translational functions. HOXA9 and HOXA13, for example, interact with the initiation factor of translation, eIF4E [22,23]. Interaction between HOXA9 and eIF4E stimulates mRNA transport by eIF4E and translation efficiency [22]. Second, Hox proteins have been involved in DNA double-stranded break (DSB) repair. HOXB7, for instance, interacts with two DNA repair proteins, Ku70/80, involved in nonhomologous end joining pathway (NHEJ) of DSB repair and confers resistance to DNA damage on irradiated cells [24]. Third, homeodomain proteins can cross biological membranes [25,26]. This feature, coupled with the possibility to be secreted into the extracellular milieu might confer to homeodomain proteins a direct cell-signalling activity. Finally, Hox proteins have been shown to play a crucial role in cell cycle regulation through the control of DNA replication. HOXD13, for example, binds DNA replication origins, primarily during G1 phase of the cell cycle, promotes the assembly of pre-replication complexes and induces DNA synthesis [27]. Such a role in cell cycle has also been suggested for HOXC10 [28] or Hoxb4, which has been associated with hematopoietic cell proliferation [29]. In this context, Hoxb4 takes part in an E3 ubiquitin ligase complex that specifically recognizes Geminin, an anti-replicative protein, and induces its degradation [30]. Therefore, it appears that Hox proteins may not simply be active as transcription factors. However, evidence about the molecular activities of Hox proteins in a non-transcriptional context remain sparse and need more extensive investigation.

Hoxa2 has been involved in rostral hindbrain and neural crest patterning. Knock-out mice for Hoxa2 display defects in hindbrain segmental identity affecting the second and third rhombomeres [31-33]. At this level, Hoxa2 has been shown to be crucial for axon guidance and the building of sensorimotor circuitry connecting the brainstem to upper nervous centres [33,34]. Hoxa2 is also essential for the identity of neural crest cells migrating from the hindbrain to the second branchial arch, which participate to the formation of skeletal derivatives notably within the middle ear [31,32].

At the molecular level, some Hoxa2 transcriptional targets such as Lmo1, Meox1, Lhx6, Ptx1, Robo2 or Six2 have been reported [35-39]. However, only Six2, Robo2 and Hoxa2 itself, have been characterized so far as direct Hoxa2 target genes [35,39-41]. A genome-wide survey of Hoxa2-bound sequences in the second branchial arch correlated to transcriptomic analyses allowed identifying large sets of candidate genes under the immediate control of Hoxa2 [18]. As interaction partners, only TALE proteins could be directly related to Hoxa2 molecular function [40] and a few candidate Hoxa2-interacting proteins have been identified mainly in the context of high-throughput experiments [42].

At the cellular level, it has been reported that Hoxa2 inhibits cell differentiation in the chondrogenic context and seems to be involved in cell migration [43-45]. Hoxa2 also inhibits differentiation of oligodendrocytes and, in addition, promotes their proliferation [46,47]. Conversely, Hoxa2 has been proposed to display an anti-proliferative activity during lung development [48]. However, the functional connection between molecular targets, cellular activities and developmental roles of Hoxa2 remains largely to be unveiled, as it is the case for other Hox proteins.

In this study, we identified three unexpected interaction partners for Hoxa2: the RING finger and CHY zinc finger domain-containing protein 1, RCHY1 (also known as PIRH2), and 20S proteasome subunits PSMA3 and PSMB2. RCHY1 is an E3 ubiquitin ligase that has been mainly involved in cell cycle and apoptosis through its activity towards the key cell cycle regulators p53 and p27Kip1 [49,50]. As a consequence of the interaction between Hoxa2 and RCHY1, we further show that Hoxa2 specifically triggers the degradation of RCHY1 in an ubiquitin-independent and proteasome-dependent manner, and in turn stabilizes p53 protein level. Together, our results show that Hoxa2 is involved in negative regulation of the RCHY1 E3 ubiquitin ligase and identify a new molecular pathway connecting Hox proteins to the p53 protein homeostasis.

## Results

### Hoxa2 interacts with RCHY1, PSMA3 and PSMB2

**Figure 1:**
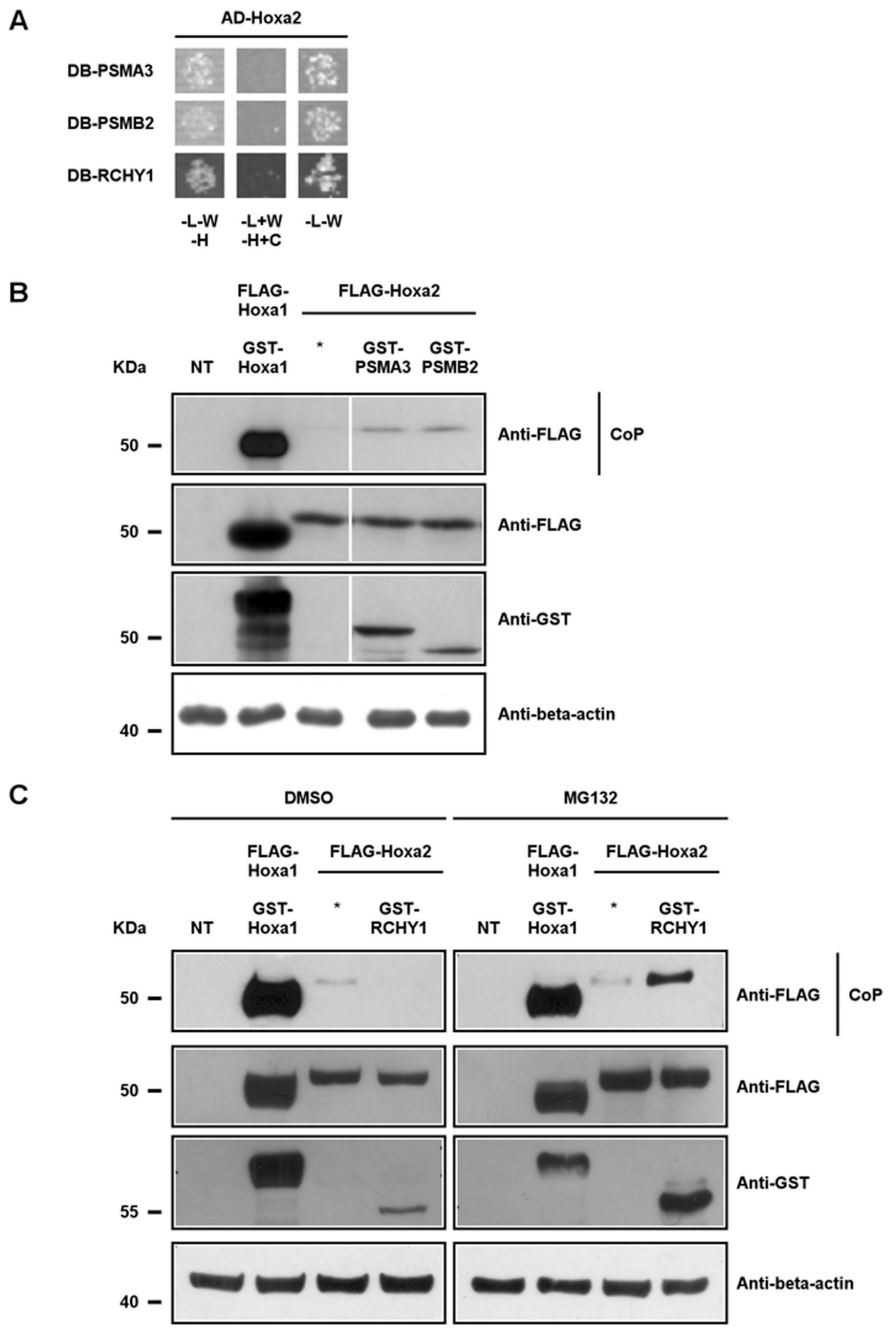
Hoxa2 interacts with RCHY1, PSMA3 and PSMB2. (A) Yeast two-hybrid analyses with Hoxa2 as prey (AD-Hoxa2) and PSMA3, PSMB2 or RCHY1 as bait (DB-PSMA3, DB-PSMB2, DB-RCHY1). Yeast transformed with expression vectors for GAL4-DB and GAL4-AD fusion proteins were mated on complete medium (YPD) and transferred on synthetic dropout medium plates lacking histidine, leucine and tryptophan (-L-W-H) to select diploids in which the GAL1-HIS3 reporter is activated as a consequence of hybrid proteins interaction. Negative control plates were composed of synthetic dropout medium containing cycloheximide and lacking histidine and leucine (-L+W-H+C) and positive controls for matings were transferred on synthetic dropout medium plates lacking leucine and tryptophan (-L-W). (B) Co-precipitation assays. HEK293T cells were co-transfected with expression vectors for FLAG- and GST-tagged Hoxa1 as a positive control, for FLAG-tagged Hoxa2 alone as a negative control and for FLAG-tagged and GST-tagged PSMA3 or GST-tagged PSMB2. Forty-eight hours after transfection, cell lysates were subjected to western blotting analyses to detect protein expression (beta-actin used as a protein load control). Protein interactions were then verified by co-precipitation on glutathione beads directed toward the GST tag. Eluted proteins were analysed by western blotting using M2 antibody to detect the presence of FLAG-tagged Hoxa2 (CoP). (C) Similar co-precipitation assays using MG132 or DMSO treated HEK293T cells reveal FLAG-tagged Hoxa2 and GST-tagged RCHY1 interaction upon proteasome inhibition.

To gain an insight into the mode of action of Hoxa2, we performed a stringent high-throughput GAL4-based yeast two-hybrid screen optimized for the testing of the entire human ORFeome [51,52]. The human ORFeome v3.1 consists of an extensive set of cloned open reading frames derived from the human genome and corresponding to 10,214 human protein coding genes [52]. Using Hoxa2 both as a bait and prey, we screened the 12,212 open reading frames (ORFs) of the human ORFeome v3.1 and identified a short isoform of the RING finger and CHY zinc finger domain-containing protein 1 (RCHY1, better known as PIRH2), the proteasome subunit alpha type-3 (PSMA3) and the proteasome subunit beta type-2 (PSMB2) as Hoxa2-interacting proteins. These results were then confirmed by retransforming expression vectors for these potential partners into yeast and retesting them against Hoxa2 (Figure 1A).

To validate these results by co-precipitation experiments in mammalian cells, we constructed expression vectors for N-terminally triple-FLAG-tagged Hoxa2 (FLAG-Hoxa2) and N-terminal glutathione S-transferase (GST)-PSMA3, -PSMB2 or -RCHY1 (full length) fusion proteins. These vectors were then co-transfected in HEK293T cells and glutathione-agarose beads were used to co-purify GST-fused interactors and FLAG-Hoxa2. As a positive control, we chose to use Hoxa1 dimer formation which had already been reported by co-precipitation [53,54] (Figure 1B). Cells transfected with FLAG-Hoxa2 alone were used as negative control. In the absence of expression vector for GST-tagged protein, no or weak background binding of FLAG-Hoxa2 was detected (Figure 1B). However, we successfully retrieved FLAG-Hoxa2 from cells co-expressing GST-PSMA3 and GST-PSMB2, therefore validating these proteins as Hoxa2 interaction partners (Figure 1B). Conversely, we failed to recover FLAG-Hoxa2 upon GST-RCHY1 co-expression (data not shown). While verifying that the fusion proteins were properly expressed in transfected cells, we were surprised to observe that, compared to cells transfected with the GST-RCHY1 vector alone, cells co-transfected with FLAG-Hoxa2 expression vector showed barely detectable weak GST-RCHY1 protein levels. This therefore indicated that expression of Hoxa2 had a negative effect on GST-RCHY1 protein accumulation. Since the GST-RCHY1 expression construct was based on a constitutively active CMV promoter, we hypothesized that the influence of Hoxa2 on the RCHY1 protein level was most likely due to an impact on RCHY1 protein stability.

### Hoxa2 induces RCHY1 degradation in a proteasome-dependent way

Since we suspected that RCHY1 protein stability was affected in presence of Hoxa2, we used a proteasome inhibitor, MG132, as the proteasome mediates one of the two main pathways of intracellular protein degradation. We exposed HEK293T cells to 1 µM of MG132, or to DMSO alone as a negative control, 24 hours after transfection, during 15 hours. In these conditions, high protein levels were observed for both FLAG-Hoxa2 and GST-RCHY1 in MG132 treated cells (Figure 1C). We next performed affinity co-purification assays using glutathione-agarose beads and validated RCHY1 as an interaction partner for Hoxa2 (Figure 1C).

**Figure 2:**
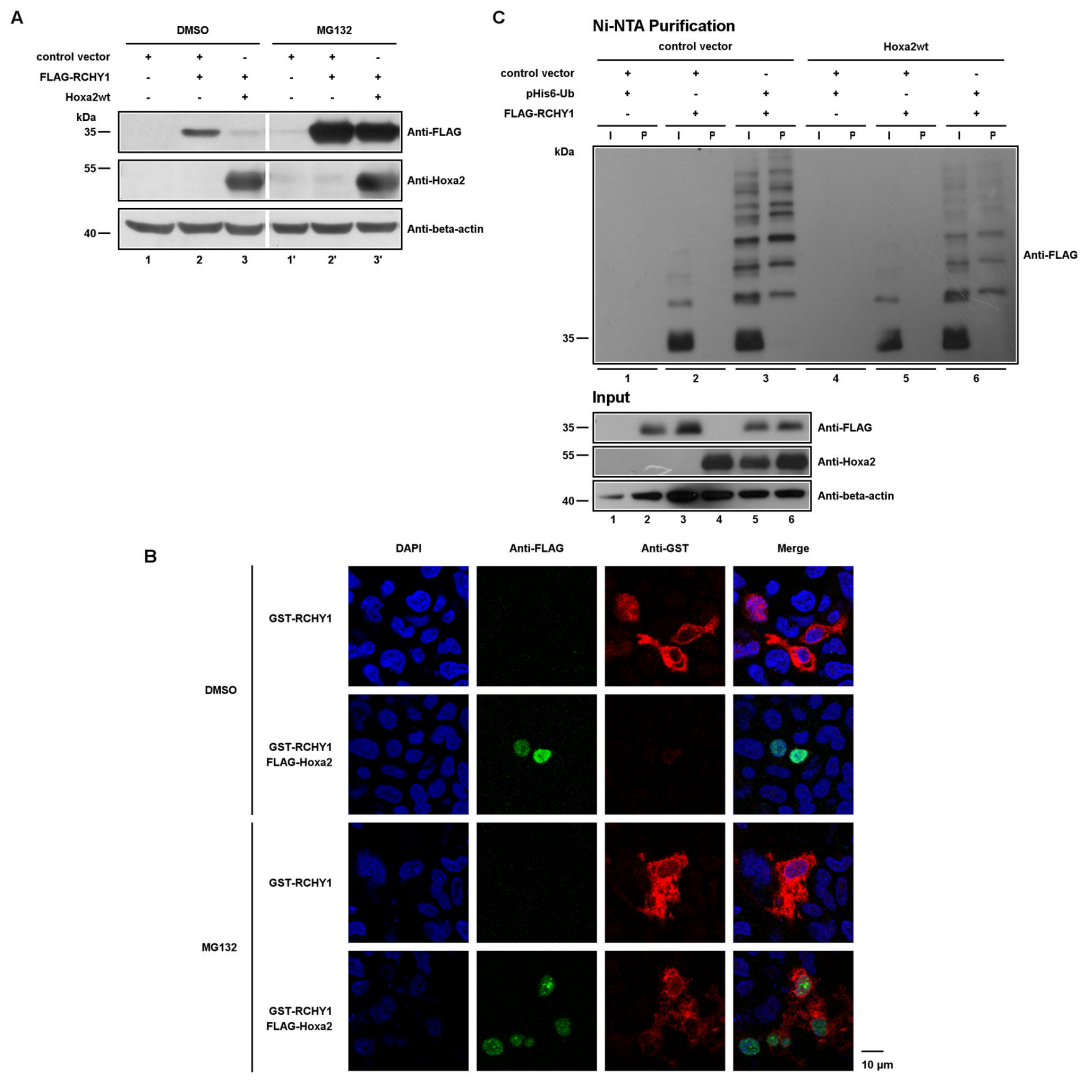
Hoxa2 induces proteasome-dependent ubiquitin-independent RCHY1 degradation and colocalizes with RCHY1 in the nucleus. (A) HEK293T cells were co-transfected with expression vectors for Hoxa2 and FLAG-tagged RCHY1, and treated with MG132 proteasome inhibitor, or DMSO. Cell lysates were loaded on SDS-PAGE for proteins separation and western blotting (beta-actin used as a protein load control). (B) FLAG-Hoxa2 and GST-RCHY1 colocalize in the nucleus. HEK293T cells were co-transfected with FLAG-tagged Hoxa2 and GST-tagged RCHY1, treated with MG132 proteasome inhibitor, or DMSO, and subjected to immunocytochemistry with anti-FLAG M2 antibody (green) and anti-GST antibody (red). Nuclei were stained with DAPI (blue). (C) Ubiquitination assays for FLAG-tagged RCHY1. HEK293T cells were co-transfected with indicated expression vectors and treated with MG132. Cells were then lysed and 6His-ubiquitin conjugated proteins were purified using Ni-NTA beads. Purified proteins and cell lysates were analysed by western blotting using M2 antibody to detect ubiquitinated forms of FLAG-tagged RCHY1. Lysate samples were loaded on a SDS-PAGE to verify protein levels prior to purification (Input; beta-actin was used as a protein load control). Lane numbering under the gels identifies cell samples. I: input sample; P: Ni-NTA purified sample.

The striking observation regarding RCHY1 protein levels in these assays focused our attention on the degradation pathway of RCHY1. To confirm the suspected involvement of Hoxa2 in RCHY1 proteasomal degradation, we co-transfected vectors for wild-type Hoxa2 and FLAG-tagged RCHY1 proteins in HEK293T. Cells were then treated with 1µM of MG132, or DMSO, during 15 hours. As shown in Figure 2A, when wild-type Hoxa2 and FLAG-tagged RCHY1 expression vectors were co-transfected, a severe depletion in FLAG-RCHY1 protein could be observed in comparison with the accumulation of FLAG-RCHY1 when it is expressed alone (Figure 2A). In addition, this Hoxa2-associated reduction in FLAG-RCHY1 protein levels was rescued by MG132 treatment thereby confirming a proteasome-dependent degradation of RCHY1 (Figure 2A). As the primary function of RCHY1 is to be an E3 ubiquitin ligase, it has also to be noticed that, contrary to what one might expect consequently to its interaction with Hoxa2, no significant decrease in Hoxa2 protein level could be observed upon co-expression with FLAG-RCHY1 (Figure 2A).

Proteasomal degradation occurs both in the nucleus and cytoplasm. As RCHY1 can be found in both subcellular compartments [55,56], the next question was to determine if we could observe a change in subcellular localization upon co-expression with Hoxa2. HEK293T cells were co-transfected with expression vectors for FLAG-Hoxa2 and GST-RCHY1 and treated with 1µM of MG132 for 15 hours. Cells were then processed to detect FLAG- and GST-tagged proteins and observed under confocal microscopy to characterize protein subcellular localization. As expected, when expressed alone GST-RCHY1 was present both in the nucleus and cytoplasm and it was more abundant when cells were treated with MG132 than in untreated cells (Figure 2B). A classical nuclear staining for Hoxa2 and a reduced staining for RCHY1 were observed in untreated cells co-expressing FLAG-Hoxa2 and GST-RCHY1 (Figure 2B). Finally, corresponding MG132-treated cells presented a nuclear co-localization for Hoxa2 and RCHY1, and no significant change in their respective intracellular distribution could be detected (Figure 2B). Since Hoxa2 and RCHY1 co-localize mainly in the nucleus, it can be assumed that their interaction, and the subsequent RCHY1 degradation, takes place in this subcellular compartment.

### Hoxa2-induced RCHY1 degradation is ubiquitin independent

As poly-ubiquitination is the main pathway for proteasomal degradation, and the only one currently known for RCHY1 degradation [49], we addressed the possibility that Hoxa2 triggers an ubiquitin-dependent RCHY1 decay. To test this, we co-transfected HEK293T cells with expression vectors for 6His-tagged ubiquitin octamer and FLAG-RCHY1, with or without expression vector for wild-type Hoxa2. Again, cells were treated with 1µM of MG132 for 15 hours to inhibit the proteasome. Protein lysates were then purified for 6His-ubiquitinated proteins using NiNTA-beads and attached proteins were then loaded on SDS-PAGE to detect RCHY1 poly-ubiquitinated forms. Unexpectedly, in the presence of Hoxa2, a reduced RCHY1 poly-ubiquitination profile was observed, indicating that the Hoxa2-induced RCHY1 degradation is ubiquitin-independent. Moreover, as less RCHY1 ubiquitinated forms were detected in the presence of Hoxa2, this suggests that the poly-ubiquitination of RCHY1 is altered upon Hoxa2 interaction (Figure 2C). These results were further confirmed with GST-RCHY1 (data not shown).

### The Hoxa2 homeodomain is essential for the Hoxa2-mediated RCHY1 degradation

**Figure 3:**
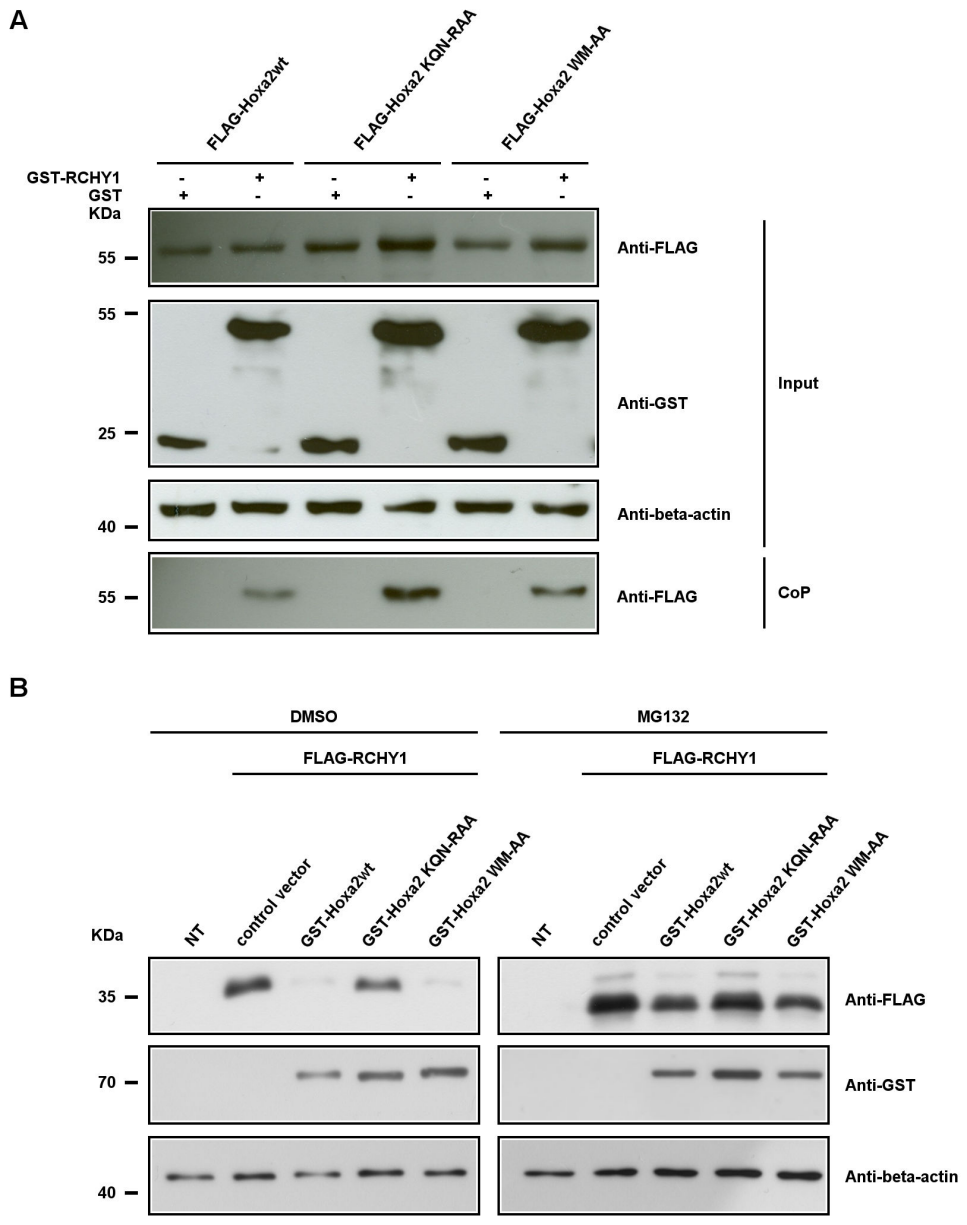
The integrity of the Hoxa2 homeodomain is required for the Hoxa2-induced RCHY1 decay. (A) Co-precipitation assays involving mutant forms of Hoxa2. HEK293T cells were cotransfected with expression vectors for FLAG-tagged Hoxa2 (FLAG-Hoxa2wt), FLAG-tagged Hoxa2^KQN-RAA^ (FLAG-Hoxa2KQN-RAA), FLAG-tagged Hoxa2^WM-AA^ (FLAG-Hoxa2WM-AA), GST-tagged RCHY1 and GST proteins, and treated with the proteasome inhibitor MG132. Forty-eight hours after transfection, cell lysates were subjected to western blotting (input) and protein interactions were verified by co-precipitation on glutathione beads directed toward the GST tag. Eluted proteins were analysed by western blotting using the M2 anti-FLAG antibody (CoP). (B) Amino acid substitutions in the Hoxa2 homeodomain abolish the Hoxa2-mediated degradation of RCHY1. HEK293T cells were transfected with expression vectors for FLAG-tagged RCHY1 (FLAG-RCHY1) and GST-tagged Hoxa2 (GST-Hoxa2wt, GST-Hoxa2KQN-RAA, GST-Hoxa2WM-AA) proteins. Cells were then treated for proteasome inhibition (MG132) and compared to untreated controls (DMSO) for the decay of FLAG-RCHY1 revealed by western blot detection. Detection of beta-actin was used as a protein load control.

To further provide mechanistic insights into the Hoxa2-mediated RCHY1 degradation, we performed experiments using mutant Hoxa2 proteins. A first Hoxa2 mutant harbours amino-acid substitutions in its homeodomain (Hoxa2^KQN-RAA^). In this mutant, the substituted glutamine and asparagine define two critical amino acid residues involved in the Hox-DNA interactions established by the third helix of the homeodomain which are shared by all Hox proteins [57]. The Hoxa2^KQN-RAA^ protein is therefore DNA-binding defective [40]. A second mutant displays substitutions in the short hexapeptide motif involved in the interaction of Hoxa2 with the Pbx proteins (Hoxa2^WM-AA^). As previously shown, these Hoxa2 variants are transcription defective [40]. Co-precipitation assays from MG132 treated HEK293T cells transfected for Flag-Hoxa2, Flag-Hoxa2^KQN-RAA^ or Flag-Hoxa2^WM-AA^ and GST-RCHY1 or a GST control revealed that both mutants were still able to bind to RCHY1 (Figure 3A). However, while Hoxa2 and Hoxa2^WM-AA^ GST fusion were prominent in inducing degradation of Flag-RCHY1, the homeodomain mutant was not. Co-expression of Flag-RCHY1 and either GST-Hoxa2 or GST- Hoxa2^WM-AA^ resulted in the disappearance of the Flag-RCHY1 in untreated cells where the proteasome was active, whereas upon expression of GST-Hoxa2^KQN-RAA^, the level of Flag-RCHY1 was unaffected (Figure 3B). 

**Figure 4:**
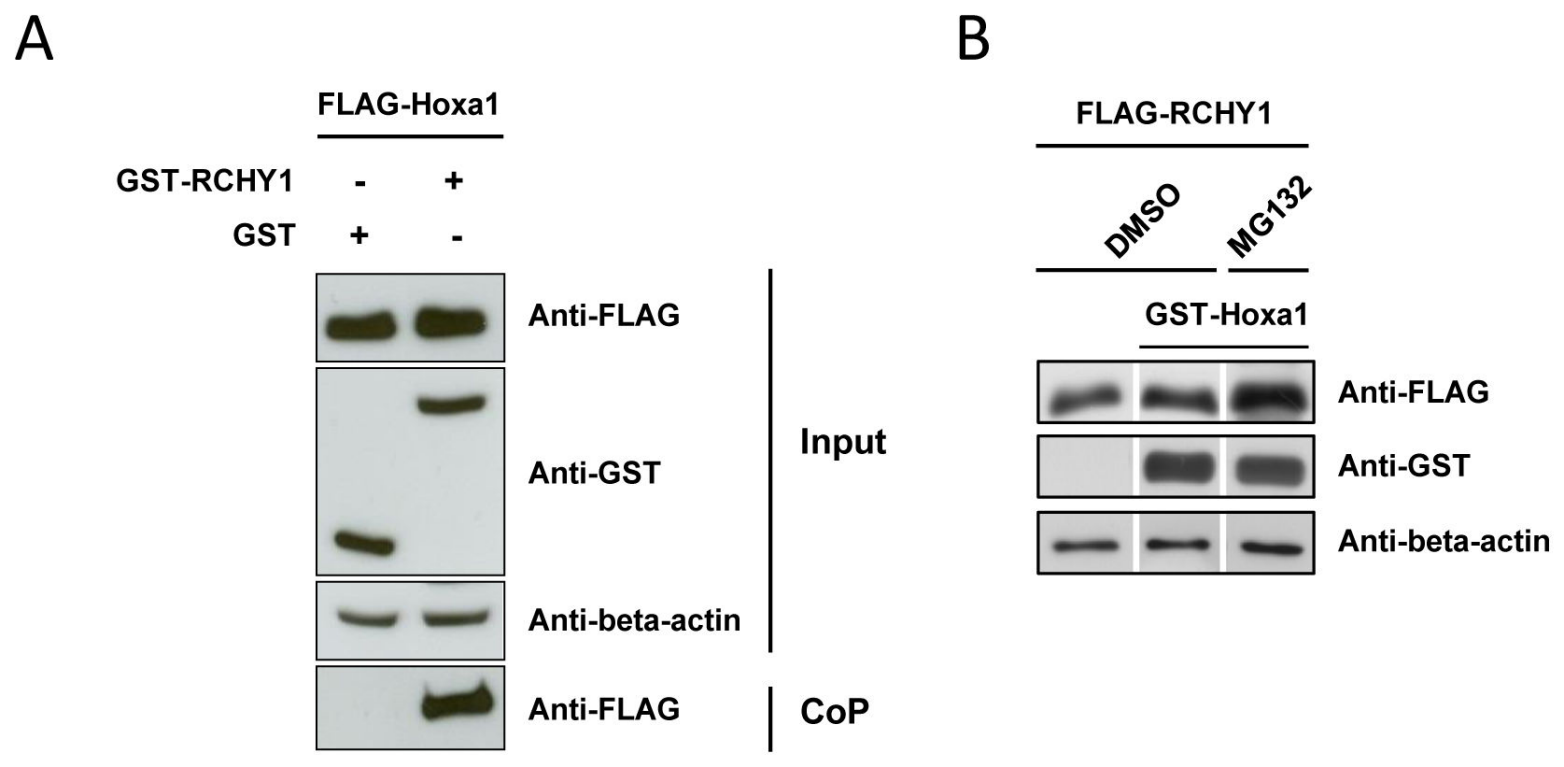
Hoxa1 does not promote RCHY1 degradation. (A) Co-precipitation assays involving Hoxa1. HEK293T cells were cotransfected with expression vectors for FLAG-tagged Hoxa1 (FLAG-Hoxa1), GST-tagged RCHY1 and GST proteins, and treated with the proteasome inhibitor MG132. Forty-eight hours after transfection, cell lysates were subjected to western blotting (input) and protein interactions were verified by co-precipitation on glutathione beads directed toward the GST tag. Eluted proteins were analysed by western blotting using the M2 anti-FLAG antibody (CoP). (B) Hoxa1 does not promote the degradation of RCHY1. HEK293T cells were transfected with expression vectors for FLAG-tagged RCHY1 (FLAG-RCHY1) and GST-tagged Hoxa1 (GST-Hoxa1) proteins. Cells were then treated for proteasome inhibition (MG132) and compared to untreated controls (DMSO) for the decay of FLAG-RCHY1 revealed by western blot detection. Detection of beta-actin was used as a protein load control.

Since Hox proteins share important sequence similarities, in particular at the level of the homeodomain, we tested whether another Hox protein, namely Hoxa1, also displayed the ability to interact with and provoke the degradation of RCHY1. Co-precipitation from HEK293T transfected cells expressing Flag-Hoxa1, and GST-RCHY1 or a GST control supported that Hoxa1 shares the ability to bind to RCHY1 (Figure 4A). Remarkably however, unlike Hoxa2, expression of a Hoxa1 fusion protein did not affect the level of RCHY1 in either MG132 or untreated cells (Figure 4B). This supports that although able to contact RCHY1, Hoxa1 is not proficient in targeting RCHY1 to proteasomal degradation. 

### Hoxa2 inhibits RCHY1-dependent ubiquitination of p53 and stabilizes p53 protein level

**Figure 5:**
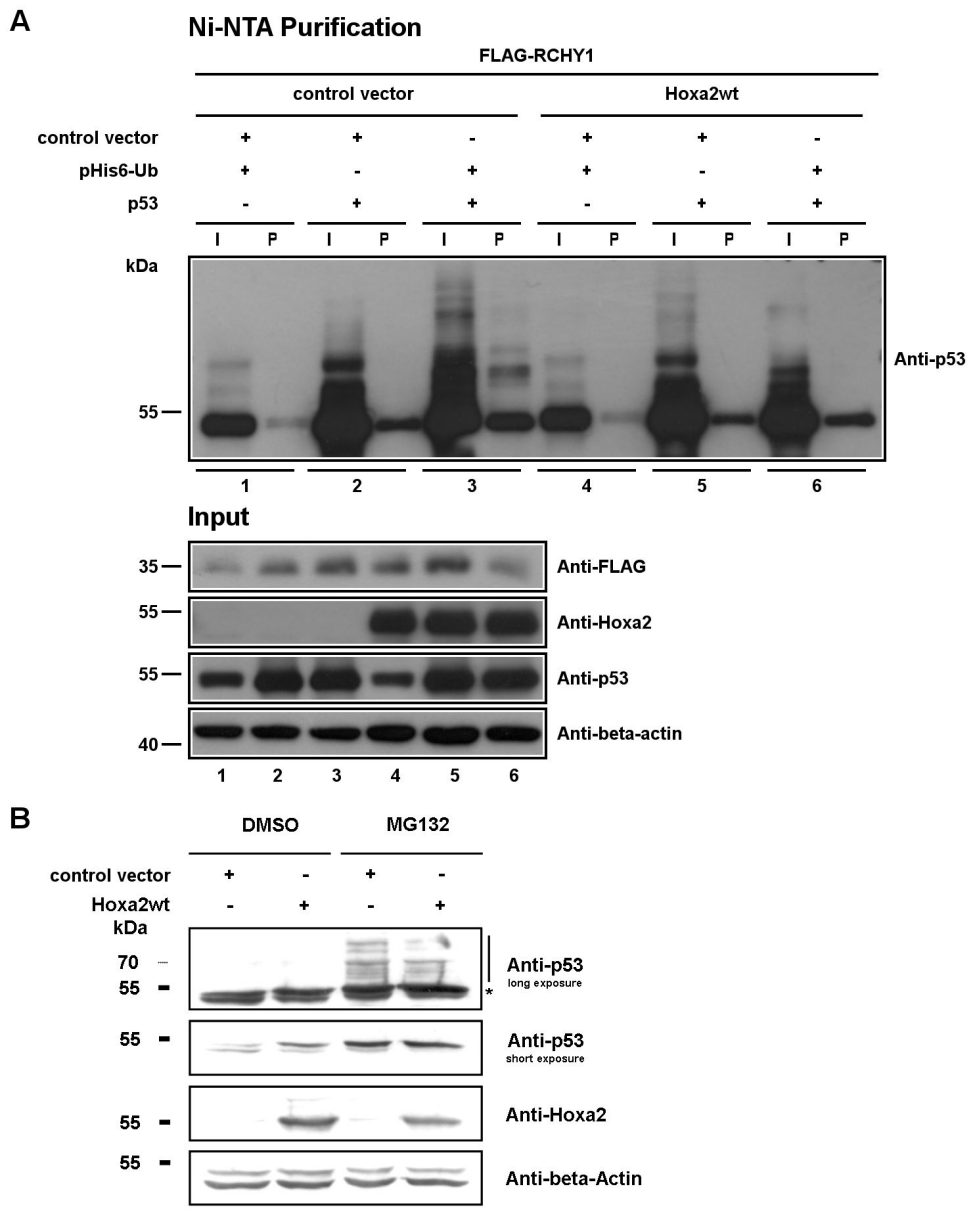
Hoxa2 inhibits RCHY1-dependent ubiquitination of p53 and stabilizes p53. (A) Ubiquitination assays for p53. HEK293T cells were co-transfected with indicated plasmids and treated with MG132. Cells were lysed and Ni-NTA beads were used to pull down 6His-ubiquitin-conjugated proteins. Proteins were separated by SDS–PAGE and western blotting was performed using an anti-p53 antibody to detect ubiquitinated forms of p53. Lysate samples were loaded on a SDS-PAGE to verify protein levels prior to purification (Input; beta-actin was used as a protein load control). Lane numbering under the gels identifies cell samples. I: input sample; P: Ni-NTA purified sample. (B) p53 protein stabilization by Hoxa2. PA1 cells were transfected with indicated plasmids and treated with MG132, or DMSO as control. Cells were lysed, proteins were separated by SDS-PAGE and western blot detection was performed with indicated antibodies.

p53 turnover has recently been revealed to occur through ubiquitination by RCHY1 and subsequent proteasomal degradation [49]. Since we observed that Hoxa2 can induce RCHY1 degradation, to get an insight into the possible biological consequences of the Hoxa2-RCHY1 interaction, we next examined the effect of Hoxa2 on p53 ubiquitination. We co-transfected cells with expression vectors for FLAG-RCHY1, p53 R72 variant (dbSNP: rs1042522) and 6His-tagged ubiquitin octamer, with or without a vector for wild-type Hoxa2. Cells were then treated with MG132 and protein lysates were purified using NiNTA-beads. Purified proteins were loaded on a SDS-PAGE gel to detect p53 poly-ubiquitinated forms. In the presence of Hoxa2, we observed less p53-ubiquitinated forms indicating that Hoxa2 negatively affects RCHY1-dependent p53 ubiquitination in MG132 treated HEK293T cells (Figure 5A). As cells were treated with MG132, these results suggest that Hoxa2 inhibits RCHY1 ubiquitin ligase activity as such. To further investigate the effect of Hoxa2 on p53 stabilization, we next transfected wild-type Hoxa2 expression vector in PA1 cells. In the presence of Hoxa2, we observed a stabilization of endogenous p53 protein level (Figure 5B). Together, these results indicate that Hoxa2 negatively affects RCHY1 stability and RCHY1-dependent ubiquitination of p53 thereby inducing a stabilization of p53 and an increase in p53 protein level.

## Discussion

While Hox proteins are well established transcription factors acting as cornerstones in the regulation of developmental processes or in some instances of oncogenesis, the molecular interactions underlying their functions have been rather poorly investigated and have been basically focused on their transcriptional activity. Nevertheless, a growing body of evidence supports that Hox proteins could be involved in other processes like translational regulation, DSB repair or DNA replication [22,24,27,30]. Here, we have characterised a new role of Hoxa2 in the proteasomal protein degradation pathway. We identified the RING finger and CHY zinc finger domain-containing protein 1, RCHY1, and two 20S core proteasome subunits, PSMA3 and PSMB2, as novel interaction partners for Hoxa2. We further showed that Hoxa2 induces proteasome-dependent RCHY1 degradation, independently of the ubiquitin system, and promotes p53 protein stabilization. We thus provide evidence for a new molecular mechanism that possibly links Hox protein activity to p53-related pathways.

Our results show that Hoxa2 interacts with RCHY1, an E3 ubiquitin ligase involved in specific degradation of key cell cycle regulators such as p53 or p27Kip1 [49,50]. This interaction promotes proteasomal degradation of RCHY1 and we provide data supporting that this degradation takes place independently of the ubiquitin system. Such a proteasomal degradation pathway independent of the ubiquitin system has already been reported for various proteins and, for some of them, the activity of the isolated 20S proteolytic core particle has been directly involved in the mechanism [58-60]. Similarly, our results show interaction between Hoxa2 and some subunits of the 20S core particle such as PSMA3, for example, which has been directly involved in the ubiquitin-independent degradation of p21Cip1 or Rb [58,61], indicating that a similar mechanism could be involved in Hoxa2-mediated degradation of RCHY1. Since we observed a nuclear co-localization of Hoxa2 and RCHY1 upon proteasome inhibitor treatment, we propose that Hoxa2 interacts with 20S core proteasome to directly induce nuclear RCHY1 proteasomal degradation in an ubiquitin-independent manner.

Distinct proteins had already been proposed to be involved in the turnover of RCHY1 [56,62,63]. However, despite its crucial role in cancer and other pathologies [64-66], only two physiological factors have been reported to negatively regulate RCHY1 at the protein level: RCHY1 itself [49] and calmodulin-dependent protein kinase II (CaMKII) [63]. Indeed, RCHY1 may be self-ubiquitinated or subject to posttranslational phosphorylation by CaMKII both leading to a decrease in RCHY1 protein levels [49,63]. Here, we thus identify Hoxa2 as a new factor involved in negative RCHY1 protein regulation. In addition to the enhanced turnover of RCHY1 by Hoxa2, we also observed a decrease in RCHY1 and p53 ubiquitination profiles in the presence of Hoxa2. This suggests that Hoxa2 interaction with RCHY1 also inhibits overall RCHY1 ubiquitin ligase activity. At the molecular level, the ability to promote RCHY1 degradation (while not its binding) relies on the integrity of the homeodomain. However, although necessary to induce RCHY1 decay, the homeodomain determinants should not be sufficient. Indeed, although sharing a highly conserved homeodomain, the Hoxa1 protein could not stimulate RCHY1 decrease. 

Our results support a model in which Hoxa2 acts as a stabilizer for p53 protein level, via its negative effect on RCHY1. This could not be expected from the known roles of Hoxa2. Indeed, p53 is known to be mainly involved in cell cycle arrest, senescence and apoptosis [67]. Thus, a p53 stabilization would, at a first glance, induce cell cycle arrest, senescence and/or increase in apoptosis, which is not in line with Hoxa2 functional studies that led to postulate anti-differentiation and pro-proliferative roles for Hoxa2 [43,46,68]. Nevertheless, besides its role in cell cycle and apoptosis, p53 has also been involved in DNA repair. The p53 response to DNA damage varies according to its subcellular localization, the cell cycle status and the extent of DNA damage, from apoptosis induction to DNA repair [69]. Efficient DNA repair is a crucial requirement during embryonic development [70]. It allows cells sustaining a high division rate, implying shorter cell cycles, but preserving quality divisions essential for developmental processes. In that context, analyses with the KEGG PathwayFinder module from the R2 microarray analysis and visualization platform (http://r2.amc.nl) allowed highlighting a wide range of transcripts positively correlated to HOXA2 expression which correspond to genes involved in DNA repair (data not shown). In support of this hypothesis, RCHY1 has been shown to induce the degradation of PolH, a member of the Y family translesion DNA polymerase involved in double-strand break repair via homologous recombination [71,72]. In addition, the study of the knock-out mouse for *Rchy1* has recently been published and confirmed its role in DNA repair [73].

Molecular activities related to such basic cell processes as DNA repair or protein turnover have already been described for Hox proteins of distinct paralog groups [24,30]. Hoxb4 has been involved in DNA replication through a direct involvement in an E3 ubiquitin ligase complex that targets Geminin for degradation [30]. HOXB7 has been directly related to DNA repair processes [24]. Considering the general involvement of Hox proteins in the control of developmental processes and their functional redundancy, it is tempting to propose that the implication of Hox proteins in DNA repair processes and protein degradation pathways could be more general than just the matter of Hoxa2. Finally, while mutant Hoxa2 proteins assayed for their ability to induce RCHY1 degradation still appear to interact with RCHY1, amino acid substitutions in the Hoxa2 homeodomain critically impairs its impact on RCHY1 turn over. A similar observation was reported for the involvement of Hoxb4 in the E3 ubiquitin ligase complex active towards Geminin. A single amino acid substitution in the Hoxb4 homeodomain was sufficient to impair its ability to enhance the poly-ubiquitination activity although the mutant protein was still able to form the complex [30]. 

## Conclusions

In conclusion, we have demonstrated that the 20S proteasome subunits PSMA3 and PSMB2 as well as the E3-ubiquitin-ligase RCHY1 are direct interactors of Hoxa2. We further showed that Hoxa2 promotes the proteasome-dependent and ubiquitin-independent degradation of RCHY1 which in turn is correlated to p53 stabilization. The RCHY1 degradation stimulated by Hoxa2 requires the integrity of its homeodomain.

## Materials and Methods

### Plasmid constructs

Expression vector for wild-type Hoxa2 was described previously [38]. Sequences coding for the wild-type Hoxa2, and mutant Hoxa2^KQN-RAA^ and Hoxa2^WM-AA^ proteins [40] were PCR-amplified and inserted into pDON223 vector using the Gateway® Technology from Invitrogen. The resulting entry plasmids were confirmed by DNA sequencing. Entry vector was then used to generate yeast expression vectors for AD- and DB-tagged Hoxa2 with pDEST-AD and pDEST-DB destination vectors (Gateway®, Invitrogen); mammalian expression vectors for FLAG-tagged Hoxa2, Hoxa2^KQN-RAA^ and Hoxa2^WM-AA^, with v1899 destination vector (for a N-terminal triple FLAG-tag fusion [74]); and mammalian expression vectors for GST-tagged Hoxa2, Hoxa2^KQN-RAA^ and Hoxa2^WM-AA^ proteins (pDEST-GST N-terminal [75]). Entry vectors for PSMA3, PSMB2 and full length RCHY1 are from the hORFeome v3.1 [76] and were used to generate destination mammalian expression vectors for N-terminal GST fusion proteins (pDEST-GST N-terminal [75]). The coding sequence for RCHY1 was also transferred into v1899 destination vector to produce a FLAG-tagged RCHY1. Expression vectors for N-terminal triple FLAG and N-terminal GST fusion Hoxa1 have been described elsewhere [54]. Expression vectors for 6His-tagged ubiquitin and p53R72 were kindly offered by Sonia Lain (University of Dundee, UK) and Patrick Dumont (Université catholique de Louvain, Belgium), respectively. In each transfection experiment, to keep the amount of transfected DNA constant, the pCAT®-Control vector (GenBank: X65321.2; Promega Corp.) coding for a Chloramphenicol acetyltransferase was invovled as a neutral control expression vector.

### Two-Hybrid screening

AD-vectors and DB-vectors were transformed into *S. cerevisiae* strains Y8800 (MATa) and Y8930 (MATα), respectively, using a one-step transformation protocol [77]. Yeast cells were plated onto synthetic dropout medium lacking tryptophan or leucine, respectively. Transformed yeasts were mated overnight at 30°C on solid medium containing yeast extract, peptone and dextrose (YEPD). Yeasts were then transferred on synthetic dropout medium plates lacking histidine, leucine and tryptophan to select diploids in which the GAL1-HIS3 reporter is activated. Control plates for autoactivation were composed of synthetic dropout medium containing cycloheximide (1 mg/L) and lacking histidine and leucine. Mating controls were plated on synthetic dropout medium lacking leucine and tryptophan.

### Cell culture and treatment

Culture cells were maintained at 37°C, in a humidified atmosphere with 5% C02. HEK293T cell line was grown in Dulbecco’s Modified Eagle Medium (D-MEM) with Gultamax-II (#31965-023, GIBCO) supplemented with 10% fetal bovine serum (#10270-106, Invitrogen), 1% penicillin-streptomycin (#15140-122, GIBCO) and 1% sodium pyruvate MEM 100 mM (#1111360-039, GIBCO). PA1 cells were maintained in Dulbecco’s Modified Eagle Medium (D-MEM) with high glucose and 25 mM HEPES (#42430, GIBCO) supplemented with 10% fetal bovine serum (#10270-106, Invitrogen) and 1% penicillin-streptomycin (#15140-122, GIBCO). For transfections, plasmid constructs were transfected with jetPRIME^TM^ transfection reagent (#114-07, Polyplus-transfection) according to the manufacturer's instructions. For proteasome inhibition, 24h after transfection, cells were treated with 10 µM MG132 in DMSO (#474790, Calbiochem), or with DMSO as control, during 15 hours.

### Protein expression analysis

Thirty-nine hours after transfection, cells were lysed for 20-30 min at 4°C in ice-cold RIPA buffer (250 mM NaCl, 50 mM TrisHCl pH7.5, 1% Nonidet P40, 0.5 % NaDeoxycholate, 0.1 % SDS, 1 mM EDTA). Lysates were then sonicated three times during 30 seconds and centrifuged. Supernatants were recovered and equal amounts of proteins (30µg) were boiled 5 minutes at 95°C in Laemmli loading buffer for SDS-PAGE (10% SDS, 50% glycerol, 250 mM Tris-HCl pH 6.8, 500 mM DTT, 0.5% bromophenol blue) and loaded on SDS-PAGE for electrophoresis. Proteins were then transferred onto a nitrocellulose membrane (Hybond™-C, #RPN303C, Amersham Biosciences). Immunoblots were performed using the SNAP i.d. protein detection system (Millipore). Membranes were blocked in 0.5% fatty acid-free BSA solution (#A6003-10G, Sigma). Anti-FLAG primary antibody (M2) (#F1804, Sigma) was used at 1:500 dilution, anti-GST primary antibody (GST-2) (#G1160, Sigma) was used at 1:300 dilution, anti-Hoxa2 primary antibody (#H9665 Sigma) was used at 1:500 dilution and anti-p53 primary antibody (Ab-6) (DO-1) (#OP43, Calbiochem) was used at 1:750 dilution. Goat anti-mouse secondary antibody was HRP conjugated and used at 1:3000 dilution (#sc-2005, Santa Cruz). For beta-actin detection, HRP conjugated anti-beta-actin was used at 1:3000 dilution (#A3854, Sigma). Finally, membranes were treated for a chemiluminescence detection system (#NEL104001EA, PerkinElmer) and exposed to a photographic film. For multiple hybridizations, membranes were rinsed 5 min in water, stripped in NaOH 0.2M for 10 min, rinsed again 5 min with water and re-analysed by western blotting.

### Protein co-precipitation

HEK293T cells were transiently transfected with vectors for FLAG-tagged-Hoxa2 variants and GST-tagged candidate interaction partners. Thirty-nine hours after transfection cells were lysed for 30 min at 4°C in ice-cold IPLS lysis buffer (0.5% NP-40, 20 mM Tris-HCl pH 7.5, 0.5 mM EDTA, 120 mM NaCl, 10% glycerol) including protease inhibitor cocktail from Roche (#11873580001, Roche). Cells lysates were centrifuged for 5 min at 16000 g at 4°C. Supernatants were recovered and samples were incubated with rotation overnight at 4 °C with glutathione-agarose beads (#G4510, Sigma) pre-washed three times with ice-cold IPLS lysis buffer. Beads were washed three times with ice-cold IPLS. Beads were supplemented with Laemmli loading buffer for SDS-PAGE (10% SDS, 30% glycerol, 350 mM Tris-Cl pH 6.8, 600 mM DTT, 0.1% bromophenol blue) and boiled 5 minutes at 95°C. Samples were centrifuged and loaded on denaturing SDS-PAGE gel for analysis by western blotting. As controls, in parallel to protein co-precipitation, expression of fusion proteins in the samples was confirmed by western blotting.

### Immunocytochemistry

HEK293T cultured on glass cover slips were transiently transfected with FLAG-tagged Hoxa2 and GST-tagged RCHY1 expression vectors and treated with MG132 proteasome inhibitor 24h after transfection. After overnight treatment, cells were rinsed in PBS solution and fixed for 30 min with 4% formaldehyde in PBS. Cells were further blocked with 10% low-fat milk in TBS-0.1% Triton X100 solution for 45 min at room temperature, followed by over-night incubation in TBS-0.1% Triton X100 solution at 4°C, with mouse anti-FLAG antibody (M2) (#F1804, Sigma) and rabbit anti-GST (#G7781, Sigma) used at 1:500 and 1:50 dilution, respectively. Cells were rinsed three times for 30 min in TBS-0.1% Triton X100 solution and incubated for 45 min at room temperature with FITC conjugated anti-mouse (#sc-3699, Santa Cruz) and TRITC conjugated anti-rabbit (#sc-2367, Santa Cruz) at 1:100 dilution in TBS-0.1% Triton X100 solution. Cells were rinsed three times and glass cover slips were mounted in Vectashield®-DAPI medium (Vector laboratories). Slides were then analysed by confocal microscopy (LSM710, Zeiss, Jena, Germany).

### Ni-NTA-resin purification for ubiquitination analyses

MG132 treated cells were lysed with imidazole containing cell lysis RIPA buffer (250 mM NaCl, 50 mM TrisHCl pH7.5, 1% Nonidet P40, 0.5 % NaDeoxycholate, 0.1 % SDS, 1 mM EDTA, 20 mM Imidazole) including protease inhibitor cocktail from Roche (#11873580001, Roche) for 20-30 min on ice. Lysates were then sonicated three times during 30 seconds and centrifuged. Supernatants were recovered and incubated with Ni-NTA Sepharose beads (#2-3201-010, Westburg) at 4°C on a rotating wheel for 2 hours. Beforehand, Ni-NTA Sepharose beads (#2-3201-010, Westburg) were pre-washed three times with ice-cold RIPA buffer (250 mM NaCl, 50 mM TrisHCl pH7.5, 1% Nonidet P40, 0.5 % NaDeoxycholate, 0.1 % SDS, 1 mM EDTA) including protease inhibitor cocktail. After incubation, beads were washed three times with imidazole containing RIPA buffer supplemented with Laemmli loading buffer for SDS-PAGE (10% SDS, 50% glycerol, 250 mM Tris-Cl pH 6.8, 500 mM DTT, 0.5% bromophenol blue). Samples were boiled 5 minutes at 95°C, centrifuged and loaded on denaturing SDS-PAGE gel for analysis by western blotting. Anti-FLAG primary antibody (M2) (#F1804, Sigma) was used at 1:500 dilution. Anti-p53 primary antibody (Ab-6) (DO-1) (#OP43, Calbiochem) was used at 1:750 dilution. Goat anti-mouse secondary antibody was HRP conjugated and used at 1:3000 dilution (#sc-2005, Santa Cruz).
